# 

**Published:** 1896-05-02

**Authors:** 


**Notices of Births, Deaths, and Marriages** are inserted in this column at a charge of 2s. 6d. for an announcement not exceeding four lines.

### NOTICE TO CORRESPONDENTS.

All MS., Letters, Books for Review, and other matters intended for the Editor should be addressed The Editor, The Lodge, Porchester Square, London, W.

The Editor cannot undertake to return rejected MS., even when accompanied by stamped directed envelope.

**Notice to Advertisers and Subscribers.**

All Advertisements, Orders for Copies of The Hospital and Business Communications should be addressed to **THE MANAGER, (Not to The Editor),** The Hospital, 428, Strand, London, W.C.

The Cost of Subscription, post free, is as follows:—Per week, 2½d.; per quarter, 2s. 8d.; per half-year, 5s. 3d.; per annum, 10s. 6d. Foreign:—Per Annum, 15s. 2d.; payable, in all cases, in advance.

Ready Shortly.

**"Burdett's Hospitals and Charities, 1896."**

Seventh year of publication. Over 1,000 pp. Scarlet cloth, gilt lettered. Price 5s. A most complete guide to British, Colonial, Indian, and American Hospitals, Dispensaries, Nursing and Convalescent Institutions, and Asylums. A few complete sets of the preceding six issues of the "Annual" may still be obtained on application to the Publishers, The Scientific Press (Limited), 428, Strand, London, W.C.

### SCRAPS AND GLEANINGS.

An honorary ophthalmic and aural surgeon has been appointed by the committee of the Wrexham Infirmary.

The sanitary alterations at the Worcester General Infirmary, which have cost nearly £1,000, have now been completed.

The York Dispensary showed an excellent report at the recent annual meeting. A balance of £289 7s. 5d. is in the hands of the treasurer.

The number of in-patients (364) and out-patients (2,863) treated during 1895 at the Kent County Ophthalmic Hospital was the largest ever recorded.

A special ward for cases of measles is supported at the Bristol Hospital for Sick Children at the sole cost of a member of the committee of that institution.

The Bethnal Green Free Library continues its useful work with increasing popularity. The Bishop of Stepney and the Rev. A. Ingram, rector of Bethnal Green, have been elected vice-presidents.

An excellent concert was recently given by the patients at the Metropolitan Convalescent Institution at Walton-on-Thames. The pianist was an inmate who has been totally blind for many years.

Owing to a very great increase in the number of cases treated during the past year at the Samaritan Hospital, Belfast, the support of the citizens is urgently solicited to enable a new wing to be added to the hospital.

The Liverpool Dramatic Society have received the thanks of the committee of the Liverpool Dental Hospital for their contribution of £33 10s. the proceeds of their entertainment given last year in aid of the institution.

The National Hospital for Consumption for Ireland will shortly be opened by the Marchioness of Zetland, but a subscription has first to be raised for the purpose of furnishing the building. The sum required is about £700.

A dinner in aid of the Morley House Convalescent Home recently took place at the Holborn Restaurant. The committee propose making extensive alterations in the home, adding a new dining-room and extra dormitories.

Much useful work has been done this year by the members of the Manchester Hospital Work Society. There are from 600 to 800 ladies in the society, each of whom contributes annually two garments for the patients in the hospitals.

The West Kent General Hospital, which has now existed for a period of 30 years, shows, by the figures presented in the annual report that a wonderful advance has been made in every branch of the institution since its commencement.

The yearly report of the Stroud Hospital shows that the subscriptions to the maintenance fund during the past year reached the sum of £310, the largest amount on record since the foundation of the hospital. The financial "equilibrium" is not, however, yet secured.

The balance sheet of the High Street Dispensary at Hastings shows receipts for the year totalling £553, with a balance in hand of £105. The report of the managing committee states that this institution maintains and carries on its useful work in a satisfactory manner.

The directors of the Aberdeen Infirmary and Asylum are endeavouring to get the roadway alongside the medical block paved with some noiseless material instead of granite sets. It is hoped in the interest of the patients that the ratepayers will not grudge the extra cost.

The meeting in connection with the Bradford Joint Hospital Fund was held last month. Efforts are to be made to obtain a contribution of a penny a month from each employé in Bradford. As the employés number 60,000 this arrangement would yield £3,000 per annum.

At the annual meeting of the governors and subscribers of the West Norfolk and Lynn Infirmary, a resolution was adopted that sufficient stock should be sold out to cover the expense of the necessary alterations and repairs to the hospital unless the money could otherwise be raised.

The committee of the Bristol Hospital for Sick Children and Women have during the past year made an important alteration in their out-patient department by the appointment of a special staff, consisting of two physicians and two surgeons to attend exclusively to that department.

A very successful social and musical evening was organised last month by the traders of Brighton on behalf of the Sussex County Hospital. The concert was held in the Dome, and half a dozen nurses made a collection in the course of the evening. The financial result amounted to about £225.

The amount received from legacies at the Chelmsford Infirmary and Dispensary has been exceptionally large, and for donations the year has been a record one, owing to the munificent gift of £1,000 from Mr. Collings West. The committee are now advising the erection of additional buildings.

April 1st (All Fools' Day) this year was turned to excellent account in the cause of charity at Thames Ditton, when a cricket match was played between some of the best known Surrey players and an eleven of comedians for the benefit of the Thames Ditton and Surbiton Cottage Hospitals. The comic entertainers appeared upon the greensward in evening dress, swallow-tails, white ties, and crush hats.

The proprietor of "Mellin's Food" has promoted another "Art Competition," with prizes to the value of £1,000. It is given under two sections, viz., the painting section, which includes original paintings in oil and water colours, and black and white sketches, and the photographic section, including almost every class of work with the camera. The exhibition will be held in January, 1897, at Queen's Hall, London, W.

Bristol, under the auspices of the present mayor and mayoress, has been considering the practicability of establishing a Hospital Saturday Fund in the town. It is hoped that the first collection will be made this year. If the effort succeeds it would be as well to consider the advisability of organising a Hospital Sunday Fund, Bristol being one of the few large towns in England without a regular Hospital Sunday or Saturday.

### BOOKS RECEIVED.

WALTER SCOTT (LIMITED).

"All-Round Cycling." By Sir B. W. Richardson and five others.

BALLIERE, TINDALL, AND COX.

"A Guide to the Practical Examination of Urine." By James Tyson, M.D. Ninth edition.

SMITH, ELDER, AND CO.

"The Treatment of Phthisis." By Arthur Ransome, M.D.

JOHN BALE AND SONS.

"Some Prolegomena to a Philosophy of Medicine." By Giles F. Goldsborough, M.D.

G. P. PUTNAM'S SONS.

"Handbook for Hospitals." By Abby Howland Woolsey, member of Committee on Hospitals, State Charities, and Association. Third edition.

CASSELL AND CO.

"Surgical Diseases of the Ovaries and Fallopian Tubes." By J. Bland Sutton, F.R.C.S. New and enlarged edition.

F. A. DAVIS CO. (Philadelphia).

"Diagnosis and Treatment of Diseases of the Rectum, Anus, and Contiguous Textures." By S. G. Gant, M.D.

J. PARKER AND CO.

"Devotional Aids to the Sick." Compiled by K. K.

**Periodicals and Pamphlets.**—London, Clinical Journal, Pediatrics, Columbus Medical Journal, Medical Press, Nursing Record, Medical Week, New York Medical Journal, Literary Digest, Charlotte Medical Journal, Therapist, Medical Pioneer, Girl's Own Paper, Leisure Hour, Boy's Own Paper, Sunday at Home, Revue Générale des Sciences, Pures, and Appliquées. The Medical Inspection of, and Physical Education in, Schools, by Chas. Roberts, F.R.C.S. (reprinted from the Report of the Royal Commission on Secondary Education, 1895. Dress and Health, by Chas. Moore Jessop, M.R.C.P.

### The Student of Medicine.

### APPOINTMENTS.

H. W. Carson, M.R.C.S., L.R.C.P., appointed Resident Medical Officer to the Evangelical Protestant Deaconess's Institution and Training Hospital, Tottenham. G. H. S. Daniell, B.A.Camb., M.B., B.C., appointed Medical Officer for the First District of the Blandford Union. R. B. Duncan, M.D.Durh., appointed Medical Officer for the Bradninch District of the Tiverton Union. J. J. Evans, M.B., M.S.Edin., appointed Senior House Surgeon to the Birmingham and Midland Eye Hospital, Birmingham. W. Galloway, L.R.C.P., L.R.C.S.Edin., appointed Medical Officer of the South Gateshead District of the Gateshead Union. D. J. Graham, appointed Medical Officer for the Orton District of the East Ward Union. F. Long, L.R.C.P.Lond., M.R.C.S.Eng., appointed Medical Officer for the Wells District of the Walsingham Union. R. S. Minnes, M.D., M.R.C.S., appointed Resident Surgical Officer to the Birmingham and Midland Eye Hospital, Birmingham. E. O. Moore, M.B., M.S.Edin., appointed Junior House Surgeon to the Birmingham and Midland Eye Hospital, Birmingham. B. B. Morton, M.B., C.M.Glasg., appointed Resident Dispensary Surgeon to the Bradford Infirmary. H. Munro, M.B., C.M.Aberd., appointed Resident Dispensary Surgeon to the Bradford Infirmary. F. S. Penny, M.R.C.S.Eng., L.R.C.P.Lond., appointed House Surgeon to the Sunderland Infirmary. H. Reeks, M.R.C.S., L.R.C.P.Lond., appointed Medical Officer of the Steyning Union Workhouse. J. Lloyd Roberts, M.D., B.S., B.A., B.Sc.Lond., F.R.C.S.Eng., appointed Assistant Honorary Physician to the Stanley Hospital, Liverpool. G. A. Simmons, M.D.Lond., appointed Medical Officer of the Fairfield House Workhouse of the Chelsea Parish.

### VACANCIES.

The following vacancies are announced this week, the amount of salary in each case being given after the name of the institution:—

### Resident Medical Officers.

Burgh of Leith.—Resident Physician to the Public Health Hospital, doubly qualified. Salary 50 guineas. Applications to T. B. Laing, Town Clerk, Town Clerk's Office, Leith, by May 9th.

Donegal District Lunatic Asylum, Letterkenny.—Assistant Medical Officer; qualified in medicine, surgery, and midwifery; unmarried, and not more than 30 years old. Salary £100 per annum, with furnished apartments, rations, washing, fuel, light, and attendance, valued at £100 per annum. Applications to Dr. Moore, Resident Medical Superintendent, by May 9th.

Hospital for Sick Children, Newcastle-on-Tyne. — Resident Medical Officer, doubly qualified. Salary £60, with board, lodging, and laundry. Applications to Robert J. Gibson, Secretary, 18, Royal Arcade, Newcastle-on-Tyne, by May 4th.

Leicester Infirmary.—Assistant House Surgeon. Appointment for six months, subject to re-election. Honorarium of £21 for the six months, with board, residence, and washing. Applications to the Secretary, 24, Friar Lane, Leicester, by April 27th. Also Honorary Ophthalmic Surgeon. Applications to the Secretary by May 4th.

Liverpool Infirmary for Children, Myrtle Street, Liverpool.—House Surgeon. Salary £85 per annum, with board and lodging. Applications to C. W. Carver, Honorary Secretary, by May 9th.

North-West London Hospital, Kentish Town Road.—Resident Medical Officer and Assistant Resident Medical Officer. Appointment for six months. Salary at the rate of £50 per annum attached to the senior post. Applications to Alfred Craske, Secretary, by May 2nd.

Royal South Hants Infirmary, Southampton.—Assistant House Surgeon. Appointment for six months. Gratuity £10. Applications to T. A. Fisher Hall, Secretary, by May 15th.

St. Marylebone General Dispensary, 77, Welbeck Street, Cavendish Square, W.—Assistant Resident Medical Officer, doubly qualified. Appointment for six months. Salary at the rate of £50 per annum, with furnished apartments, attendance, coal, and light. Applications to the Secretary by May 4th.

Stockport Infirmary.—Junior Assistant House Surgeon. Appointment for six months, with board and residence. Honorarium of £10. Applications to the Secretary by May 5th.

### Non-Resident Medical Officers.

Borough of Portsmouth.—Medical Officer of Health, Port Sanitary Medical Officer, and Medical Officer to the Infectious Diseases Hospital. Must devote his whole time to the duties. Salary £450 per annum, rising to £550 per annum as a maximum by four annual instalments. Applications endorsed "Medical Officers' Application" to be sent to Alexander Hellard, Town Clerk, by May 2nd.

Dental Hospital of London, Leicester Square, W.—Anæsthetist and Assistant Anæsthetist. Must be duly-qualified medical practitioners. Applications to J. Francis Pink, Secretary, by May 11th.

Dental Hospital of London and London School of Dental Surgery, Leicester Square.—Lecturer on Mechanical Dentistry. Applications to Morton Smale, Dean, by May 11th.

Hastings, St. Leonards, and East Sussex Hospital. — Honorary Ophthalmic Surgeon. Applications to the Secretary at the Hospital, White Rock, Hastings, by May 2nd.

Metropolitan Hospital, Kingsland Road, N.E.—Surgeon and an Assistant Surgeon. Must be F.R.C.S.Eng. Applications to C. H. Byers, Secretary, by May 4th.

Plymouth Royal Eye Infirmary.—Honorary Surgeon. Applications to the Secretary by May 9th.

